# Perfluorinated Chemicals and Fetal Growth: A Study within the Danish National Birth Cohort

**DOI:** 10.1289/ehp.10506

**Published:** 2007-08-16

**Authors:** Chunyuan Fei, Joseph K. McLaughlin, Robert E. Tarone, Jørn Olsen

**Affiliations:** 1 Department of Epidemiology, University of California, Los Angeles, California, USA; 2 International Epidemiology Institute, Rockville, Maryland, USA; 3 Vanderbilt University Medical Center, Vanderbilt–Ingram Cancer Center, Nashville, Tennessee, USA; 4 Institute of Public Health, Aarhus University, Aarhus, Denmark

**Keywords:** birth weight, cord blood, length of gestation, low birth weight, maternal blood, PFOA, PFOS, preterm birth, small for gestational age

## Abstract

**Background:**

Perfluorooctanesulfonate (PFOS) and perfluorooctanoate (PFOA) are man-made, persistent organic pollutants widely spread throughout the environment and human populations. They have been found to interfere with fetal growth in some animal models, but whether a similar effect is seen in humans is uncertain.

**Objectives:**

We investigated the association between plasma levels of PFOS and PFOA in pregnant women and their infants’ birth weight and length of gestation.

**Methods:**

We randomly selected 1,400 women and their infants from the Danish National Birth Cohort among those who completed all four computer-assisted telephone interviews, provided the first blood samples between gestational weeks 4 and 14, and who gave birth to a single live-born child without congenital malformation. PFOS and PFOA were measured by high performance liquid chromatography–tandem mass spectrometer.

**Results:**

PFOS and PFOA levels in maternal plasma were on average 35.3 and 5.6 ng/mL, respectively. Only PFOA levels were inversely associated with birth weight (adjusted β = −10.63 g; 95% confidence interval, −20.79 to −0.47 g). Neither maternal PFOS nor PFOA levels were consistently associated with the risk for preterm birth or low birth weight. We observed no adverse effects for maternal PFOS or PFOA levels on small for gestational age.

**Conclusion:**

Our nationwide cohort data suggest an inverse association between maternal plasma PFOA levels and birth weight. Because of widespread exposure to these chemicals, our findings may be of potential public health concern.

Perfluorooctanesulfonate (PFOS) and perfluorooctanoate (PFOA) are persistent organic pollutants (POPs), which are produced synthetically or from the metabolism of other perfluorinated chemicals (PFCs) [Butenhoff et al. 2006; U.S. Environmental Protection Agency (EPA) 2006]. These compounds are widely used as industrial surfactants and emulsifiers and in numerous consumer products. Nonstick pans, carpets, furniture, household cleaners, shampoos, shoes, clothing, and convenience food packaging are some of the products that can contain PFCs (Butenhoff et al. 2006; U.S. EPA 2006).

PFOA and PFOS are slowly metabolized and have half-lives in humans of around 4 years and 5 years, respectively (Olsen et al. 2007). They are absorbed through oral intake or inhaled and, to a lesser extent, through dermal exposure. They have been detected in the sera of occupationally and nonoccupationally exposed human populations in various countries (Calafat et al. 2007; Kannan et al. 2004; Karrman et al. 2006; Olsen et al. 2003; Yeung et al. 2006), including pregnant women and their newborn babies (Apelberg et al. 2007a; Inoue et al. 2004; Jin et al. 2004; Karrman et al. 2007; Midasch et al. 2007; So et al. 2006; Tittlemier et al. 2004).

Exposure of rodent dams during pregnancy to both PFOS and PFOA has been associated with decrements in birth weight and length of gestation at birth (Butenhoff et al. 2004b; Case et al. 2001; Grasty et al. 2003; Lau et al. 2004, 2006; Luebker et al. 2005a, 2005b; Thibodeaux et al. 2003; Wolf et al. 2007). Although it is unknown how PFOS and PFOA may impair fetal growth, several mechanisms have been suggested, including a disturbance of lipid metabolism (Kennedy et al. 2004; Luebker et al. 2005b), a reduction of food and water intake, or altered hormones levels (Betts 2007; Lau et al. 2004; Luebker et al. 2005b; Thibodeaux et al. 2003), but a direct fetotoxic effect has also been suggested (Betts 2007; Henderson and Smith 2007; Hinderliter et al. 2005; Luebker et al. 2005a, 2005b). No effect of PFOS exposure on fetal growth in humans was found in a study based on self-reports of birth weight and occupational histories (Grice et al. 2007) or in a study of maternal and cord blood samples from 15 children (Inoue et al. 2004). However, a recent study reported levels of both PFOS and PFOA to be inversely associated with birth weight, newborn head circumference, crown–heel length, and ponderal index, but the study was based on cord blood samples taken after delivery (Apelberg et al. 2007b). No large-scale study based on prospectively collected biomarkers at the time of fetal growth has been published to date.

In this study we examined the relation between maternal plasma PFOS and PFOA levels during pregnancy and birth weight as well as length of gestation using data from the Danish National Birth Cohort (Olsen et al. 2001).

## Methods

### Study population

The Danish National Birth Cohort (DNBC) includes data on 91,827 pregnant women from March 1996 to November 2002 (Olsen et al. 2001). Women were recruited nationwide (a population of 5.4 million people) by their general practitioners (GPs) early in pregnancy. About half of all GPs in the country participated, and about 60% of the pregnant women accepted the invitation to participate. After providing written informed consent, women took part in four computer-assisted telephone interviews at approximately 12 and 30 weeks of gestation, and at approximately 6 and 18 months after birth. A food frequency questionnaire was filled out at home approximately at gestational week 25. Moreover, blood was drawn twice from the women, once in the first and once in the second trimester. An umbilical cord blood sample was obtained shortly after birth. Each blood sample was sent by ordinary mail to the State Serum Institute in Copenhagen, Denmark, and then separated and stored in freezers at −20°C or in liquid nitrogen. Blood samples were thus transported at outside temperatures for 4–48 hr, but most of the samples arrived within 28 hr.

Among all participants who gave birth to a single live-born child without a reported congenital malformation (*n* = 87,752), who provided the first blood sample between gestational weeks 4 and 14 (*n* = 80,678), and who had responded to all four telephone interviews (*n* = 43,045), we randomly selected 1,400 mothers. Of the 1,400 selected, 1,102 had a second blood sample, and 200 were randomly selected among these. Of these 200, 146 had a cord blood sample from the child, and 50 were randomly selected among these. We used the second maternal and cord blood samples to examine whether PFOS and PFOA levels were stable during pregnancy and whether the mother–offspring correlation in blood levels was high. Sample sizes were determined in large part by the capacity of the laboratory to perform PFOS and PFOA assays and by what would be acceptable for the Steering Committee that gives permission for use of cohort data.

Information on potential confounders—such as infant sex, maternal age, parity, socio-occupational status, prepregnancy body mass index (BMI), and smoking during the pregnancy—was collected by highly structured questionnaires (available at http://www.bsmb.dk) (Olsen et al. 2001). Socio-occupational status was based on education and current job titles. Women with a higher education (4 years beyond secondary school education) or in management level jobs were classified as “high” social status; women with middle-range training and skilled workers were classified as “middle”; and unskilled workers and unemployed were classified as “low.” Birth weight and gestational age at birth were obtained from the National Hospital Discharge Register at the National Board of Health in Denmark. The assessment of gestational age was done by the midwives based on either the first day of the last menstrual period (LMP) (conditioned on a regular bleeding pattern during the preceding 6 months, and no use of oral contraceptives during the 3 months preceding pregnancy) or from ultrasound examination done before 24 weeks of gestation (Albertsen et al. 2004). If estimates differed, ultrasound measures replaced the LMP estimates. In most cases, gestational age was based on early ultrasound measures. If data were missing or out of the expected range (*n* = 10), we used the expected date of delivery provided by the pregnant women at the second interview (after their ultrasound examinations). Low birth weight (LBW) was defined as birth weight < 2,500 g. Preterm birth was defined as the birth of an infant before 37 completed weeks of gestation (259 days), post-term birth as after 42 completed weeks of gestation (294 days), and term birth as between 37 and 42 completed weeks of gestation. Small for gestational age (SGA) was defined as an infant with birth weight below the 10th percentile at a specific gestational age in weeks, based on all singleton live births of same sex and parity who fulfilled our sampling criteria.

This research was approved by the UCLA Office for Protection of Research Subjects (Reference No. 06-08-023-01) and the Danish Data Protection Agency (Reference No. J.Nr 2006-41-6324).

### Exposure assessment

We measured plasma concentrations of PFOS and PFOA using high performance liquid chromatography–tandem mass spectrometry in the 3M Toxicology Laboratory (Ehresman et al. 2007). The laboratory was blinded to birth outcomes and types of blood drawn (first or second maternal blood, or cord blood). Stable labeled analogs of PFOS (^18^O_2_ PFOS) and PFOA (^13^C_2_ PFOA) were used in all extracted procedures. All extractions were performed using solid phase extraction techniques and based on 100 μL of plasma. Details of the analytical methods used are available elsewhere (Ehresman et al. 2007).

Quality control materials, a single lot of newborn calf serum, were analyzed together with the samples to ensure the accuracy and reliability of the data. Previous evaluation comparing serum and plasma samples has shown no significant difference when using an extracted serum spiked standard curve (Ehresman et al. 2007). Two levels of spiked calf serum controls were extracted using 30 individual solid phase extractions to establish “within”-run means and standard deviations for each separate level of control. These levels were calculated at 15 ng/mL and 44 ng/mL for PFOA and 10 ng/mL and 30 ng/mL for PFOS. A total of 35 batch runs was necessary to complete all sample analyses. The coefficient of variations for the between-batch spiked control values were 3.5% and 3.2% for PFOA, respectively, and 2.8% and 2.5% for PFOS, respectively. Blind samples were also selected and relabeled for repeated analysis. There were strong correlations between the repeated blind analyses and the original results for PFOS (*r* = 0.993, *n* = 100) and PFOA (*r* = 0.987, *n* = 100). Only two PFOA values and no PFOS values were reported at the lower limit of quantitation (LLOQ) of 1.0 ng/mL. Values below the LLOQ (*n* = 2) were assigned a value of half the LLOQ. PFOS and PFOA measurements were determined on the residual plasma component from 15 frozen whole blood samples (12 first maternal blood samples, 1 second blood sample, and 2 cord blood samples). These were multiplied by a factor of two to make them comparable to measurements made on plasma (Ehresman et al. 2007).

### Statistical analysis

We used analyses of variance and linear regression to assess the associations between birth weight and length of gestation and maternal plasma PFOS and PFOA levels. We used logistic regression to estimate odds ratio (OR) and 95% confidence interval (CI) for low birth weight, SGA, and preterm birth. The PFOS and PFOA levels were entered into the analyses as both continuous and categorical variables (< 25th, 25th–< 50th, 50th– < 75th, ≥ 75th). Plasma levels below the 25th percentile were used as the reference group when using categorical variables. Both log-transformed and untransformed blood PFC concentrations were tested in the statistical models, and the findings were similar. For this article, we used only the results of untransformed concentrations. We also conducted regression diagnostics for influential points using Cook’s distance, but no substantial changes in estimates were observed before or after the influential points were excluded, and our results are therefore all based on the whole data set.

We adjusted for risk factors that could influence fetal growth or length of gestation at birth. These factors included maternal age, parity, socio-occupational status, prepregnancy BMI, smoking during pregnancy, infant sex, and gestational week at blood drawing. For birth weight, we also adjusted for gestational age at birth, measured in days and entered as linear and quadratic terms into the model to capture the reduced weight gain during the last weeks. All covariates but gestational age at birth and gestational week at blood drawing were introduced into models as indicator variables. Stratified analyses by parity, prepregnancy BMI, term of birth (preterm, term, and post-term) and sex were also performed to evaluate the consistency of the associations across strata.

## Results

Demographic and other characteristics of the study participants are presented in Table 1. Almost half of the women were having their first babies, and the average age at delivery was about 30 years. Almost 25% smoked in early pregnancy, but one-third of these reported quitting smoking during pregnancy. Almost 30% had a prepregnancy BMI of ≥ 25 kg/m^2^. The mean birth weight was 3,623 g, and the incidence of LBW was 1.7%; the mean gestational age at birth was 280 days, and the incidence of preterm birth and post-term birth was 3.8% and 9.9%, respectively (Table 2). On average, birth weight increased with maternal age, parity, and prepregnancy BMI and was lower among smokers (Table 1).

All the first maternal blood samples had PFOS levels above the LLOQ, and only one had a PFOA level below the LLOQ. The plasma concentrations varied substantially, with a range of 6.4–106.7 ng/mL for PFOS values, and less than the LLOQ to 41.5 ng/mL for PFOA values. PFOS and PFOA levels decreased with increasing parity. The mean concentration of PFOA was highest in the age group of < 25 years (6.2 ng/mL), and lowest in the age group of ≥ 35 years (5.1 ng/mL), but after adjustment for parity the difference was reduced to 0.4 ng/mL. High levels were also observed in overweight and obese women (Table 1).

**Figure 1:**
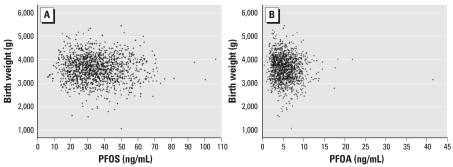
Birth weight and plasma PFOS (*A*) and PFOA (*B*) concentrations in the first maternal blood samples.

Figure 1 shows scatterplots for the first maternal blood PFOS and PFOA levels and birth weight. The adjusted association between PFOS levels and birth weight was close to null, but birth weight was inversely associated with plasma levels of PFOA in the first blood sample (Table 3). The unadjusted regression coefficient was −20.52 (95% CI, −31.49 to −9.56) (Figure 1), but adjustment for potential confounders reduced the estimate to −10.63 (95% CI, −20.79 to −0.47) (Table 3). Parity was the covariate that most markedly changed the regression coefficient. PFOA levels were correlated with PFOS levels (*r* = 0.62), and after adjustment for PFOS levels in the first maternal blood, the regression coefficient of PFOA became slightly stronger (β = −14.85; 95% CI, −27.67 to −2.02). Analyses stratified by term of birth (preterm, term, post-term) revealed that the effects of PFOA on birth weight were more pronounced in preterm and post-term babies (Table 3), but did not differ markedly by sex or parity. PFOA levels were significantly inversely associated with birth weight only among normal-weight women.

Infants born to mothers with PFOA levels in the three highest quartiles had an adjusted average birth weight of 96, 98, and 105 g lower than infants born to mothers in the lowest quartile, respectively (Table 4). No statistically significant association was observed between either PFOS or PFOA and length of gestation (Table 4). Elevated risk estimates for preterm birth were observed at all levels of PFOS and PFOA, but only the ORs for the third quartile of PFOS and the second quartile of PFOA were statistically significant (Table 5). We did not observe any statistically significant association between LBW or SGA and maternal PFOS or PFOA levels (Table 5).

**Figure 2:**
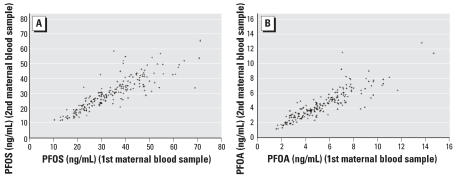
Plasma PFOS (*A*) and PFOA (*B*) concentrations between the first and second maternal blood samples.

**Figure 3:**
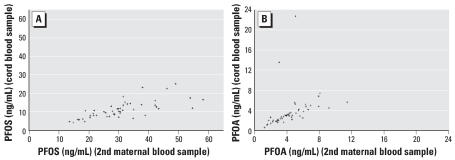
Plasma (*A*) and PFOA (*B*) concentrations between the second maternal and cord blood samples.

There was a high degree of correlation for PFOS or PFOA levels between the first and second maternal blood samples (*r* = 0.87 for PFOS and 0.88 for PFOA) (Figure 2), but the average concentrations were lower in the second blood samples. PFOS and PFOA concentrations in cord and maternal blood were also correlated (*r* = 0.72 for PFOS and 0.84 for PFOA if two outliers were not included) (Figure 3), but cord levels were significantly lower than maternal levels. The mean ratios between first maternal PFOS and PFOA and cord concentrations were, respectively, 3.40 and 1.83, but decreased between second maternal and cord concentrations to 2.96 and 1.46.

## Discussion

To our knowledge, this is the first nationwide population-based study on birth weight and length of gestation with prospectively collected data on levels of perfluorinated chemicals in maternal sera drawn early in pregnancy. We found only maternal plasma PFOA levels to be inversely associated with birth weight. Risk estimates for preterm birth did not increase monotonically across quartiles of PFOS or PFOA exposure. For all four outcomes evaluated, the outcome in the lowest exposure quartile differed from outcomes in the upper three quartiles, but there was no evidence of a dose–response association above the 25th percentile.

Maternal plasma levels in the present study (mean concentrations: PFOS, 35.3 ng/mL; PFOA, 5.6 ng/mL) are similar to most levels reported for U.S. populations, but much lower than those found in fluorochemical workers or residents living near the fluoropolymer production facilities (Emmett et al. 2006a, 2006b; Olsen et al. 2003). We found PFOS and PFOA levels higher than those seen among pregnant women in other countries (Inoue et al. 2004; Karrman et al. 2007; Midasch et al. 2007), but that may reflect blood drawing at different gestational week, or use of different assay laboratory technologies.

This study has several strengths. We used state-of-the-art laboratory facilities, rigorous blinding, and hospital records for obtaining data on birth weight and gestational age. We found a high correlation between the concentrations of PFOS and PFOA taken over pregnancy time, but the levels declined over pregnancy time, possibly related to blood volume expansion and decreased serum albumin concentration during pregnancy (Swanson and King 1983; Thibodeaux et al. 2003), changes in PFC disposition and pharmacokinetics during pregnancy (Frederiksen 2001), or placental transfer of PFCs to the fetus (Apelberg et al. 2007a; Inoue et al. 2004; Midasch et al. 2007; Tittlemier et al. 2004). We also found a high correlation between maternal and cord blood concentrations. In this study we used only one measurement of PFOS and PFOA, and it is unknown how well this marker reflects an etiologically relevant dose, which may be the average dose over the entire pregnancy duration, a concentration at a different time in pregnancy, or the concentration in fetal blood. These concentrations do vary with gestational age, but whether this is important for a potential fetotoxic effect is not known.

Our nationwide study has the advantage of being selected from a well-described cohort of > 90,000 pregnant women and their infants (Olsen et al. 2001). Selection bias related to PFOA and PFOS levels or birth weight is unlikely, because participants were enrolled early in pregnancy and had no information on any of these variables at that time. Information on gestational age and birth weight came from the Danish Hospital Discharge Register and was based on standard clinic procedures using ultrasound based estimates and electronic weights. We also benefited from extensive data on potential confounders. Besides the covariates in our final model, we evaluated possible confounding by alcohol drinking, fish intake, protein, fat, carbohydrate, and energy intake. Data were missing on dietary covariates for around 20% of births, but the results remained materially the same after these adjustments.

Adjusting for parity and prepregnancy BMI substantially changed the crude measures of association. For instance, adjustment for only parity in the regression model led to attenuation of the regression coefficient between PFOA and birth weight from −20.5 to −7.2. We did find substantially higher PFOA levels in nulliparous women than in multiparous women, which has been shown before in cord blood (Apelberg et al. 2007a), perhaps related to fetal uptake during pregnancy and excretion during lactation.

Unlike the more lipophilic POPs, PFOS and PFOA do not typically accumulate in the lipid tissues because of their oil-repellent properties (Martin et al. 2003). Because these compounds are retained primarily in blood and liver, PFOS and PFOA would not be expected to be associated with BMI. Our data did, however, demonstrate higher plasma levels of PFOS and PFOA among overweight or obese women. In a study among 3M male employees (Olsen et al. 1998), BMI was also slightly but not significantly higher in the highest PFOA category. In stratified analyses, PFOA levels were only significantly associated with birth weight in normal-weight women, suggesting effect-measure modification by prepregnancy BMI of the association between PFOA and birth weight.

PFOS and PFOA can cross the placental barrier, but cord concentrations are usually lower than those observed in maternal serum or plasma (Inoue et al. 2004; Midasch et al. 2007; Tittlemier et al. 2004). In a recent study based on 11 plasma samples of mothers and the 11 corresponding cord plasma samples of neonates (Midasch et al. 2007), PFOS cord concentrations were significantly lower than the maternal values, but 9 of 11 neonates had higher PFOA plasma level compared with those of their mothers. We found lower mean concentrations of PFOA and PFOS in cord blood than in maternal blood, but PFOA levels in cord blood were much closer than PFOS to maternal values, which may indicate that PFOA crosses the placental barrier more easily than PFOS. If so, that may partly explain why we observe only an inverse association between PFOA and birth weight, although animal studies, as well as one cord blood study in humans (Betts 2007), indicate an effect of both chemicals.

The association between PFOA and birth weight may or may not have public health importance, even if it is causal. LBW has been associated with higher mortality and morbidity that is not restricted to early years of life (Glucksman and Hanson 2006), although this may reflect underlying causes of fetal growth impairment rather than LBW itself. Furthermore, the observed association may not be causal. We did not find a monotone dose–response relation, but our results for birth weight, risk of preterm delivery, LBW, and SGA are consistent with a threshold effect. Chance may play a role in some of our findings, and it is also possible that there are factors associated with both lowest PFOA levels and high birth weight that were not taken into account in our statistical analyses.

The incidence of LBW and preterm birth in our study population is relatively low due to the selection criteria used for sampling the sub-cohort. This should be taken into consideration when trying to extrapolate our results to other populations. We used clinical recordings of gestational age that were often ultrasound-based. Ultrasound-based estimates are biased if the exposure of interest reduces very early fetal growth. This may explain to some extent the higher incidence of preterm birth we see among the exposed, but analysis of gestational age by exposure category did not provide evidence for such an effect, and such a bias would not explain differences in mean birth weight.

We observed an inverse association between PFOA levels and birth weight, although the maternal concentrations were orders of magnitude lower than those at which developmental effects have been observed in the animal toxicity studies. A risk characterization of PFOA shows average sera concentrations associated with the lower 95% confidence limit of the benchmark dose for 10% response levels for various developmental outcomes were at least 29,000 ng/mL in rat (Butenhoff et al. 2004a). The approximate maternal sera concentrations at which a significant reduction of fetal body weight occurred in mice were much higher, at 175,000 ng/mL (Lau et al. 2006). Contrary to the relatively rapid rates of elimination reported in laboratory animals, PFOA is eliminated slowly in humans (Kennedy et al. 2004). Therefore, it is hard to make a comparison with animal data, and the necessary toxicokinetic data in humans are lacking. Still, the adverse effect on birth weight observed at low levels of exposure in our study combined with ubiquitous PFOA contamination in human blood may be a cause for concern.

In conclusion, our nationwide cohort study suggests an inverse association between maternal levels of PFOA and birth weight. Although PFOS and PFOA have been phased out by some manufacturers in the United States and in some European countries, they are still produced elsewhere, and other similar compounds are believed to break down to PFOS or PFOA in the environment (Organisation for Economic Co-operation and Development 2002; U.S. EPA 2006). If these compounds reduce fetal growth, continued exposure will be of public health concern, and other potential reproductive effects should be studied.

## Figures and Tables

**Table 1:** Plasma concentrations of PFOS, PFOA, and birth weight (mean ± SD) by characteristics of study subjects (*n* = 1,400).

Characteristic^a^	No. (%)	PFOS (ng/mL)	PFOA (ng/mL)	Birth weight (g)
Maternal age at delivery (years)				
< 25	118 (8.4)	38.6 ± 12.0	6.2 ± 2.1	3,573 ± 492
25–29	547 (39.1)	36.8 ± 12.8	6.0 ± 2.8	3,590 ± 521
30–34	504 (36.0)	33.9 ± 13.2	5.2 ± 2.2	3,676 ± 519
≥ 35	230 (16.4)	33.0 ± 12.7	5.1 ± 2.4	3,629 ± 581
Parity				
0	626 (44.7)	37.7 ± 13.0	6.6 ± 2.7	3,524 ± 514
1	508 (36.3)	33.2 ± 12.7	4.7 ± 1.9	3,689 ± 501
2	225(16.1)	34.0 ± 12.6	4.8 ± 2.3	3,723 ± 580
≥ 3	41 (2.9)	30.5 ± 11.7	3.7 ± 1.6	3,862 ± 540
Socio-occupational status				
High	709 (50.8)	34.0 ± 12.7	5.6 ± 2.3	3,648 ± 527
Middle	566 (40.5)	36.6 ± 12.9	5.6 ± 2.8	3,603 ± 531
Low	121 (8.7)	36.5 ± 14.1	5.6 ± 2.3	3,606 ± 536
Prepregnancy BMI (kg/m^2^)				
< 18.5	58 (4.2)	33.1 ± 14.3	5.2 ± 2.2	3,396 ± 539
18.5–24.9	905 (66.2)	34.6 ± 12.9	5.5 ± 2.6	3,620 ± 506
25.0–29.9	299 (21.9)	36.3 ± 12.0	5.6 ± 2.3	3,638 ± 547
≥ 30.0	105 (7.7)	39.3 ± 14.4	6.1 ± 2.7	3,770 ± 542
Smoking during the pregnancy				
Nonsmoker	1,052 (75.1)	35.7 ± 13.3	5.6 ± 2.6	3,661 ± 514
Quit smoking	131 (9.4)	33.9 ± 11.6	5.8 ± 2.2	3,700 ± 592
1–9 cigarettes/day	109 (7.8)	35.5 ± 12.7	5.8 ± 2.6	3,434 ± 469
≥ 10 cigarettes/day	108 (7.7)	32.5 ± 11.9	4.9 ± 1.9	3,384 ± 560
Sex				
Female	690 (49.3)	35.3 ± 13.0	5.5 ± 2.4	3,582 ± 538
Male	710 (50.7)	35.2 ± 12.9	5.6 ± 2.7	3,668 ± 518

a Missing data: maternal age (1), socio-occupational status (4), prepregnancy BMI (33), birth weight (12).

**Table 2:** Concentrations of PFOS and PFOA in the maternal and cord blood samples and birth outcomes of study subjects.

	No.	Value
1st maternal blood levels (ng/mL)^a^		
PFOS	1,399	35.3 ± 13.0
PFOA	1,399	5.6 ± 2.5
2nd maternal blood levels (ng/mL)		
PFOS	200	29.9 ± 11.0
PFOA	200	4.5 ± 1.9
Umbilical cord blood levels (ng/mL)		
PFOS	50	11.0 ± 4.7
PFOA	50	3.7 ± 3.4
Birth weight (g)	1,388	3623 ± 238
Low birth weight (< 2,500 g)		24 (1.7)
Gestational age (days)	1,399	280.4 ± 11.0
Preterm birth (< 259 days)		53 (3.8)
Post-term (≥ 294 days)		139 (9.9)

Values are mean ± SD or no. (%).

a A total of 1,399 first maternal blood samples were analyzed because one blood sample was missing.

**Table 3:** Adjusted regression coefficients [β (95% CI)] between PFOS and PFOA (ng/mL) in first maternal blood during pregnancy and birth weight (g).

Strata	PFOS	PFOA
All^a^	−0.46 (−2.34 to 1.41)	−10.63 (−20.79 to −0.47)
Term of birth^a^		
Preterm	−2.27 (−20.94 to 16.40)	−43.38 (−141.11 to 54.34)
Term	0.29 (−1.72 to 2.27)	−8.73 (−19.53 to 2.06)
Post-term	−4.82 (−11.10 to 1.46)	−24.49 (−58.04 to 9.06)
Parity^b^		
Nulliparous	−1.17 (−4.40 to 2.06)	−10.94 (−24.00 to 2.13)
Multiparous	−1.23 (−3.82 to 1.35)	−14.84 (−30.95 to 1.28)
Sex^c^		
Female	−1.85 (−4.49 to 0.79)	−11.45 (−26.76 to 3.85)
Male	1.11 (−1.56 to 3.79)	−10.36 (−24.07 to 3.34)
Prepregnancy BMI (kg/m^2^)^d^		
< 18.5	5.42 (−4.34 to 15.17)	26.09 (−48.06 to 100.23)
18.5–24.9	−1.45 (−3.76 to 0.85)	−15.75 (−27.75 to −3.75)
25.0–29.9	0.52 (−3.96 to 4.99)	−2.03 (−26.91 to 22.85)
≥ 30.0	−0.33 (−6.60 to 5.94)	1.64 (−33.11 to 36.39)

a Adjusted for gestational age, quadratic gestational age, infant sex, maternal age, socio-occupational status, parity, cigarette smoking, prepregnancy BMI, gestational weeks at blood drawing. The category definitions of the covariates were the same as shown in Table 1.

b The models did not include parity.

c The models did not include sex.

d The models did not include prepregnancy BMI.

**Table 4:** Birth weight (g) and gestational age (days) by PFOS and PFOA (in quartiles) in first maternal blood during pregnancy.

	Birth weight (g)			Gestational age (days)		
	No.	Unadjusted mean	SD	No.	Unadjusted mean	SD
PFOS (ng/mL)						
6.4–26.0	347	3,640	519	349	281.0	9.7
26.1–33.3	346	3,673	539	348	280.4	10.8
33.4–43.2	346	3,590	506	349	279.6	10.8
≥ 43.3	348	3,602	552	352	280.4	11.4
PFOA (ng/mL)						
< LLOQ–3.91	348	3,737	531	349	281.1	9.6
3.91–5.20	346	3,611*	539	349	280.0	11.3
5.21–6.96	349	3,595**	512	351	279.7	11.2
≥ 6.97	344	3,562**	524	349	280.8	11.7

Because of missing records (1), blood sample (1), and birth weight (12), only 1,387 observations were used in this analysis. Due to missing records or those records with the same values at cut-off points, the numbers in each quartile are not equal.

* Statistically significantly different from the lowest quartile, *p*-value < 0.01.

** Statistically significantly different from the lowest quartile, *p*-value < 0.001.

**Table 5:** Adjusted OR (95% CI) for preterm birth, LBW, and SGA according to PFOS and PFOA levels (in quartiles) in the first maternal blood during pregnancy.

	Preterm birth^a^		LBW^a^		SGA^b^	
	No.	OR (95%CI)	No.	OR (95%CI)	No.	OR (95%CI)
PFOS (ng/mL)						
6.4–26.0	7	1.00 (reference)	2	1.00 (reference)	36	1.00 (reference)
26.1–33.3	14	2.30 (0.86–6.12)	5	3.39 (0.37–31.16)	26	0.73 (0.42–1.26)
33.4–43.2	21	2.83 (1.10–7.30)	9	6.00 (0.73–49.34)	25	0.68 (0.39–1.19)
≥ 43.3	11	1.43 (0.50–4.11)	8	4.82 (0.56–41.16)	34	0.98 (0.58–1.65)
Trend (*p*-value)^c^		0.79		0.13		0.88
PFOA (ng/mL)						
< LLOQ–3.91	6	1.00 (reference)	2	1.00 (reference)	29	1.00 (reference)
3.91–5.20	18	2.94 (1.05–8.28)	7	4.27 (0.50–36.51)	33	1.17 (0.68–2.00)
5.21–6.96	17	2.51 (0.87–7.27)	8	3.73 (0.42–32.45)	29	1.06 (0.61–1.84)
≥ 6.97	12	1.71 (0.55–5.28)	7	2.44 (0.27–22.25)	30	0.97 (0.55–1.70)
Trend (*p*-value)^c^		0.55		0.94		0.81

a Adjusted for infant sex, maternal age, socio-occupational status, parity, prepregnancy BMI, gestational weeks at blood drawing, and cigarette smoking during pregnancy.

b Adjusted for maternal age, socio-occupational status, prepregnancy BMI, gestational weeks at blood drawing, and cigarette smoking during pregnancy.

c The categories of PFOS or PFOA levels were entered as continuous variables in the test for trend.
